# Dosimetric comparison of inverse optimisation methods versus forward optimisation in HDR brachytherapy of breast, cervical and prostate cancer

**DOI:** 10.1007/s00066-019-01513-x

**Published:** 2019-09-03

**Authors:** Georgina Fröhlich, Gyula Geszti, Júlia Vízkeleti, Péter Ágoston, Csaba Polgár, Tibor Major

**Affiliations:** 1 Centre of Radiotherapy, National Institute of Oncology, Ráth György Street 7–9, 1122 Budapest, Hungary; 2 Faculty of Science, Eötvös Loránd University, Pázmány Péter mall 1/A, 1117 Budapest, Hungary; 3 Faculty of Medicine, Department of Oncology, Semmelweis University, Ráth György Street 7–9, 1122 Budapest, Hungary

**Keywords:** Inverse optimisation algorythms, HIPO hybrid inverse planning optimisation, IPSA inverse planning simulated annealing, Breast brachytherapy, Prostate brachytherapy, Cervical brachytherapy, Inverse Optimierung algorithmus, HIPO hybrid inverse planning optimisation, IPSA inverse planning simulated annealing, Mamma-Brachytherapie, Prostata-Brachytherapie, Zervix-Brachytherapie

## Abstract

**Objective:**

Dosimetric comparison of HIPO (hybrid inverse planning optimisation) and IPSA (inverse planning simulated annealing) inverse and forward optimisation (FO) methods in brachytherapy (BT) of breast, cervical and prostate cancer.

**Methods:**

At our institute 38 breast, 47 cervical and 50 prostate cancer patients treated with image-guided interstitial high-dose-rate BT were selected. Treatment plans were created using HIPO and IPSA inverse optimisation methods as well as FO. The dose–volume parameters of different treatment plans were compared with Friedman ANOVA and the LSD post-hoc test.

**Results:**

IPSA creates less dose coverage to the target volume than HIPO or FO: V100 was 91.7%, 91% and 91.9% for HIPO, IPSA and FO plans (*p* = 0.1784) in breast BT; 90.4%, 89.2% and 91% (*p* = 0.0045) in cervical BT; and 97.1%, 96.2% and 97.7% (*p* = 0.0005) in prostate BT, respectively. HIPO results in more conformal plans: COIN was 0.72, 0.71 and 0.69 (*p* = 0.0306) in breast BT; 0.6, 0.47 and 0.58 (*p* < 0.001) in cervical BT; and 0.8, 0.7 and 0.7 (*p* < 0.001) in prostate BT, respectively. In breast BT, dose to the skin and lung was smaller with HIPO and FO than with IPSA. In cervical BT, dose to the rectum, sigmoid and bowel was larger using IPSA than with HIPO or FO. In prostate BT, dose to the urethra was higher and the rectal dose was smaller using FO than with inverse methods.

**Conclusion:**

In interstitial breast and prostate BT, HIPO results in comparable dose–volume parameters to FO, but HIPO plans are more conformal. In cervical BT, HIPO produces dosimetrically acceptable plans only when more needles are used. The dosimetric quality of IPSA plans is suboptimal and results in unnecessary larger active lengths.

## Zusammenfassung

**Ziel:**

Es erfolgte ein dosimetrischer Vergleich von inversen und Vorwärtsoptimierungsmethoden (FO) aus dem Bereich HIPO („hybrid inverse planning optimization“) und IPSA („inverse planning simulated annealing“) in der Brachytherapie (BT) des Mamma‑, Zervix- und Prostatakarzinoms.

**Methoden:**

In die Studie wurden 38 Mamma-, 47 Zervix- und 50 Prostatakarzinompatienten aufgenommen, die mit bildgesteuerter interstitieller hochdosierter BT behandelt worden waren. Behandlungspläne wurden mittels inversen sowie Vorwärtsoptimierungsmethoden aus dem Bereich HIPO und IPSA erstellt. Die Dosisvolumenparameter der verschiedenen Behandlungspläne wurden durch Friedman-ANOVA („analysis of variance“) und auf die LSD („least significant difference“) bezogene Post-hoc-Tests miteinander verglichen.

**Ergebnisse:**

Bei IPSA wird die Zielvolumenabdeckung durch den Einsatz geringerer Dosis im Vergleich zu HIPO oder FO erreicht; V100 war 91,7%, 91% und 91,9% für HIPO-, IPSA- und FO-Pläne (*p* = 0,1784) bei Mamma-BT; 90,4%, 89,2% und 91% (*p* = 0,0045) bei Zervix-BT sowie 97,1%, 96,2% und 97,7% (*p* = 0,0005) bei Prostata-BT. Durch HIPO entstehen gleichmäßigere Pläne, der COIN („conformal index“) betrug 0,72; 0,71 und 0,69 (*p* = 0,0306) bei Mamma-BT; 0,6; 0,47 und 0,58 (*p* < 0,001) bei Zervix-BT sowie 0,8; 0,7 und 0,7 (*p* < 0,001) für Prostata-BT. Bei Mamma-BT war die erfasste Dosis in Haut und Lungen mit HIPO und FO niedriger als mit IPSA. Im Rahmen der Zervix-BT war die Dosis in Rektum, Sigma und Darm bei Nutzung von IPSA erhöht im Vergleich zu HIPO oder FO. Bei FO-Anwendungen war die Dosis bei Prostata-BT im Vergleich zu den inversen Methoden an der Urethra erhöht, die Rektaldosis dagegen erniedrigt.

**Schlussfolgerung:**

Bei der interstitiellen Mamma- und Prostata-BT erreicht HIPO vergleichbare Dosisvolumen-Parameterwerte wie FO, die HIPO-Pläne sind jedoch deutlich konformaler. Bei Zervix-BT generiert HIPO dosimetrisch akzeptable Pläne nur bei Verwendung von mehreren Nadeln. Die dosimetrische Qualität von IPSA-Plänen ist suboptimal und führt zu unnötiger zusätzlicher Bestrahlung.

## Introduction

In spite of some early publications on inverse optimisation in brachytherapy (BT) [1, 2], inverse dose planning has played a significant role in external beam radiotherapy (EBRT) treatment planning since 2000 [3]. In brachytherapy these methods have become widespread in the past decade [4]. Beside the reproducibility of the plans, their practical advantage is reduced planning time. However, it needs the accurate setup of the initial preset and it is beneficial only if sufficient degrees of freedom are available for the algorithm. Therefore, inverse optimisation works only for interstitial BT treatments, where a large number of needles and source positions are available. Although, the planning time is shorter than with forward optimisation methods, more volumes (i.e. organs at risk) are generally needed. For example, automatic skin contouring is appropriate for forward optimisation (FO) in interstitial breast BT, but a special 5 mm thick skin shell is needed for inverse methods. Even though inverse methods are currently generally used in several types of interstitial BT, only a few studies with a small number of patients have been conducted on this [5–11]. Comprehensive dosimetric evaluation and comparison with the forward method are still awaited.

Several inverse methods were developed during the past decade, but only two are applied widely. Hybrid inverse planning optimisation (HIPO) is a heuristic, hybrid deterministic stochastic dose–volume-based inverse optimisation method [12]. The stochastic algorithm, called simulated annealing, searches the optimal catheter distributions for a given set of dose objectives. The deterministic algorithm, called dose–volume histogram-based optimisation, optimizes 3D dose distribution quickly by moving straight downhill once it is in the advantageous region of the search space given by the stochastic algorithm. For optimisation of the dwell times of the radioactive source, the limited-memory Broyden–Fletcher–Goldfarb–Shanno (L-BFGS) quasi-Newtonian algorithm is used. The given dose–volume constraints are reached at the same time with the minimalisation of several cost functions.

Inverse planning simulated annealing (IPSA) is a heuristic stochastic anatomy-based inverse optimisation method [13]. It is determined by the cost function, which represents the dose prescription and constraints. Since it was implemented for low-dose-rate seed prostate treatments, it optimises the dwell positions of the source, too. Both HIPO and IPSA inverse optimisation algorithms have been implemented in Elekta BT treatment planning products (Elekta Brachytherapy, Veendendaal, the Netherlands).

The aim of present study is to analyse the dosimetric effect of HIPO and IPSA inverse optimisation algorithms and compare it to the forward optimisation method in high-dose-rate interstitial BT treatments of breast, cervical and prostate cancer.

## Materials and methods

At our institute, 38 breast, 47 cervical and 50 prostate cancer patients treated with image-guided high-dose-rate interstitial BT were selected for this study.

Postoperative multicatheter breast BT implantation was performed using a preimplant CT image set. Based on the postimplant CT, the planning target volume (PTV) and organs at risk were created and the treatment plan was normalised to the basal dose points using the optimal value of the F‑factor and graphical and manual optimisation [11] were used (Oncentra Brachy v4.5.3, Elekta Brachytherapy, Veendendaal, the Netherlands). The prescribed dose was 30.1 Gy in 7 fractions, twice a day. The detailed description of our treatment method can be found in our previous publication [14].

BT boost treatment for cervical cancer was delivered with a combined interstitial intracavitary technique, given 1 or 2 fractions weekly. Patients were treated with 4 BT fractions of 7 Gy. Initial and post-teletherapy MRI were used to determine the number and position of needles in the ring- or Fletcher-type interstitial applicator. The implantation was transrectal US guided. The delineation of PTV, bladder, rectum, sigmoid and bowel was performed on postimplant CT, also using information from post-teletherapy MRI. During treatment planning, graphical and manual optimisation was used to achieve an optimal dose distribution (Oncentra Brachy v4.5.3, Elekta Brachytherapy, Veendendaal, the Netherlands). The detailed description of our treatment method can be found in our previous publications [11, 15].

Transrectal US-guided transperineal interstitial prostate implantation was performed during the 4 weeks of the EBRT course in a single fraction. After scanning the prostate with US, a virtual preimplant plan was generated (Oncentra Prostate v3.1, Elekta Brachytherapy, Veendendaal, the Netherlands). The HIPO optimisation method was used, and the prescribed dose was 10 Gy to the whole prostate gland (V100 ≥ 95%). Based on this plan, metal needles were inserted into the prostate through a template under live US guidance. The optimisation procedure was used again for the dwell times in the inserted needles to achieve the final dose distribution. The detailed description of our treatment method can be found in our previous publication [16].

Patients were treated with a high-dose-rate (HDR) remote afterloading unit (microSelectron v3, Elekta Brachytherapy, Veendendaal, the Netherlands) using an Ir-192 stepping source (type v2) with an initial contained activity of 370 GBq (reference air kerma rate of 40.7 mGy * m^2^/h). The used source step was 2.5 mm.

**Fig. 1:**
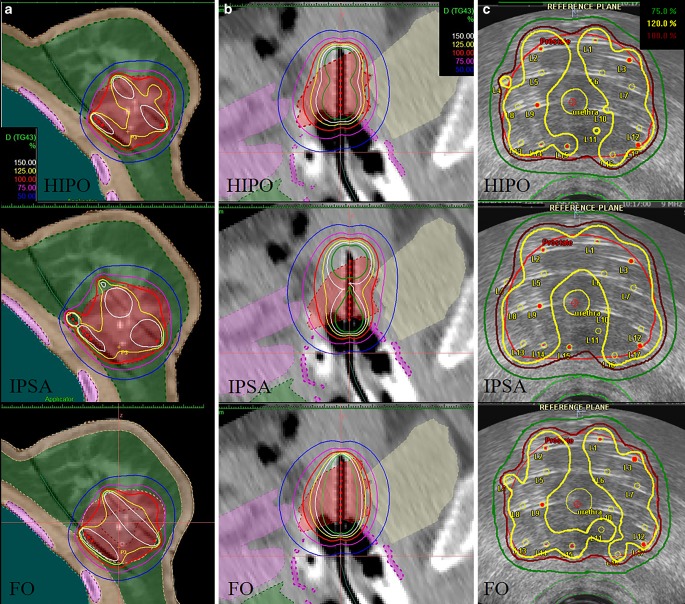
Dose distributions using HIPO (hybrid inverse planning optimisation), IPSA (inverse planning simulated annealing) and forward optimisation (*FO*) in interstitial BT (brachytherapy) of breast (**a**), cervical (**b**) and prostate (**c**) cancer. *Red dots: *active dwell positions (volumes: *red: *PTV [planning target volume]; **a** *green:* non-target breast, *blue: *ipsilateral lung, *pink: *ribs; **b** *yellow:* bladder, *green:* rectum, *violet:* sigmoid, *pink: *vagina; **c** *yellow:* urethra, *green: *rectum)

Additional treatment plans were created using HIPO and IPSA inverse or forward (graphical and manual) optimisation methods (Fig. 1). To avoid inter-observer variations, only one physicist who is well-experienced in interstitial BT made all the plans. With both inverse methods, our library preset was used first (the used constraints are in Tables 4 and 5 in the Appendix), then this initial preset was modified to achieve the optimal dose distribution in each plan. It has to be noted that inverse algorithms can plan the optimal catheter distribution only in the Oncentra prostate planning system. In breast and cervical BT, this feature is not needed, as the dose planning process is not in real-time. We defined the catheter positions in a simple manual way before implantation and did not use this automatic option in prostate planning neither [11, 14–16]. With FO and HIPO, active dwell positions were inside or on the surface of the PTV, while IPSA optimised the source dwell positions and created active dwells outside the PTV, too. Using HIPO, the recommended 0.2 value of the dwell time gradient restriction was used to modulate the ratio of dwell times of the adjacent dwell positions [17].

The following dose–volume parameters were used for quantitative evaluation of the plans:

- *V100, V150:* the volume of the PTV receiving 100% and 150% of the prescribed dose (%) [18],
- *V*_*100*_*, V*_*150*_*, V*_*PTV*_: absolute volume irradiated by 100% and 150% of the prescribed dose (cc) and the volume of the PTV,
- *D90*: the minimum dose delivered to 90% of PTV (Gy) [19],
- *DNR*: dose nonuniformity ratio [20, 21]
- *DHI*: dose homogeneity index [22, 23],
- *COIN*: conformal index [24],
- *D*_*2*_*(x)*: the minimal dose to the most exposed 2 cc of *the critical organ x* (% or Gy) [25],
- *D*_*1*_*(x)*: the minimal dose to the most exposed 1 cc of *the critical organ x* (%),
- *D*_*0.1*_*(x)*: the minimal dose to the most exposed 0.1 cc of *the critical organ x* (%),
- *V50(x)*: absolute volume to *the critical organ x* irradiated by 50% of the prescribed dose (cc), where x is *non-target breast, contralateral breast, skin, lung, heart, bladder, rectum, sigmoid or bowel*.

The dose–volume parameters do not follow the Gaussian distribution (F-tests were significant for all parameters), so the studied parameters of different treatment plans were compared with non-parametric Friedman ANOVA and the LSD post-hoc test (Statistica 12.3, StatSoft, Tulsa, OK, USA).

## Results

Both HIPO and IPSA methods decrease the time of planning process to FO, from 25 to 15 min in breast BT, from 10 to 7 min in cervical BT and from 20 to 10 min on average in prostate BT.

IPSA created less dose coverage to the target volume than HIPO or FO, V100 was 91.7%, 91% and 91.9% for HIPO, IPSA and FO plans (*p* = 0.1784) in breast BT, 90.4%, 89.2% and 91% (*p* = 0.0045) in cervical BT and 97.1%, 96.2% and 97.7% (*p* = 0.0005) in prostate BT, respectively.

**Fig. 2:**
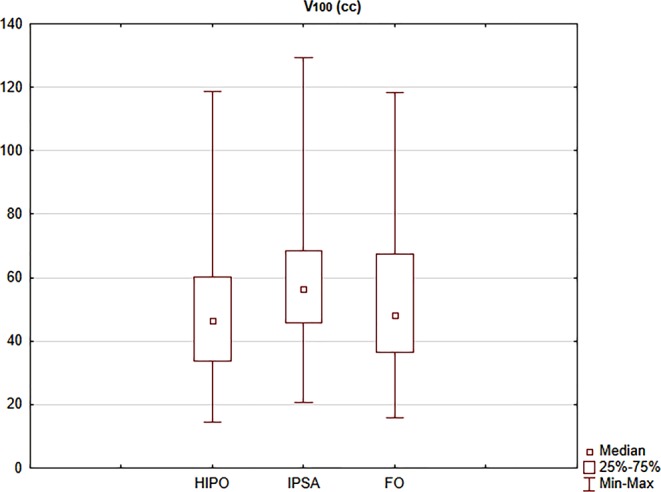
The absolute volume irradiated by 100% of the prescribed dose (*V*_*100*_) using HIPO (hybrid inverse planning optimisation), IPSA (inverse planning simulated annealing) and forward optimisation (*FO*) methods in interstitial cervical BT (brachytherapy) plans

In cervical BT plans, IPSA created larger volumes irradiated by the prescribed dose, V_100_ was 48.5 cc, 59.6 cc and 52.8 cc in HIPO, IPSA and FO plans, respectively (*p* < 0.001; Fig. 2). The volumes irradiated with high doses were also larger in IPSA plans: V_150_ was 26.7 cc, 30.3 cc and 28.9 cc (*p* < 0.001), respectively. In the case of breast and prostate BT plans, there were no significant differences in these parameters (*p* = 0.0806 and 0.1038).

In breast BT, all of the plans were appropriately homogeneous, DNR was 0.3, 0.3 and 0.29 in HIPO, IPSA and FO plans, respectively (*p* = 0.1524). In the case of cervical BT, IPSA resulted in the most homogeneous plans, with DNR values of 0.55, 0.50 and 0.54 (*p* < 0.001), respectively. HIPO plans were more homogeneous in prostate BT, DHI was 0.7, 0.6 and 0.6 (*p* < 0.001).

HIPO resulted in more conformal plans, COIN was 0.72, 0.71 and 0.69 (*p* = 0.0306) in breast BT, 0.6, 0.47 and 0.58 (*p* < 0.001) in cervical BT and 0.8, 0.7 and 0.7 (*p* < 0.001) in prostate BT, respectively.

In breast BT, the dose to the skin and lung was smaller with HIPO and FO than with IPSA. D_1_(skin) was 19.8%, 21.8% and 17.1% (*p* = 0.0425), D_0.1_(lung) was 42.7%, 57.4% and 44.7% (*p* = 0.0457) in HIPO, IPSA and FO plans, respectively. In cervical BT, the dose to the rectum, sigmoid and bowel was larger using IPSA. D_2_(rectum) was 2.5 Gy, 2.7 Gy and 2.6 Gy (*p* = 0.0009), D_2_(sigmoid) was 3.2 Gy, 3.6 Gy and 3.1 Gy (*p* < 0.001) and D_2_(bowel) was 4.1 Gy, 4.6 Gy and 4.2 Gy (*p* = 0.0049), respectively. In prostate BT, the dose to the urethra was higher in FO plans than in inverse optimised plans, D_0.1_(urethra) was 113.8%, 112.6% and 124.6% (*p* < 0.001), respectively. However, the rectal dose was smaller using FO, D_2_(rectum) was 57.4%, 59.2% and 50.5% (*p* < 0.001), respectively.

The detailed statistical data are in Table 1 for breast, Table 2 for cervical and Table 3 for prostate BT plans.

**Table 1:** Dose–volume parameters (mean and range, median) in interstitial breast BT using HIPO (hybrid inverse planning optimisation), IPSA (inverse planning simulated annealing) and forward optimisation (FO)

Dose–volume parameter	HIPO	IPSA	FO	*p*-value (ANOVA)^b^	*p*-value (post hoc)^b^
Nr. of needles	13.5 (7–28)^a^			–	–
V_PTV_ (cc)	60.2 (26.9–173.6)			–	–
V100 (%)	91.7 (87.6–96.4)	91.0 (88.8–98.3)	91.9 (90.0–96.0)	0.1784	–
V150 (%)	35.5 (25.8–50.9)	33.2 (16.1–60.3)	36.0 (23.0–42.5)	*0.0205*	HIPO-IPSA: 0.0409 IPSA-FO: 0.0169
DNR	0.30 (0.25–0.45)	0.30 (0.20–0.50)	0.29 (0.25–0.37)	0.1524	–
COIN	0.72 (0.50–0.80)	0.71 (0.50–0.80)	0.69 (0.49–0.82)	*0.0306*	HIPO-FO: 0.0339 IPSA-FO: 0.0493
V50(non-target breast, cc)	0.8 (0.3–1.2)	1.3 (0.5–1.7)	0.9 (0.3–1.5)	*0.0027*	HIPO-IPSA: 0.0245 IPSA-FO: 0.0436
D_1_(contralateral breast, %)	1.4 (0.1–1.9)	1.6 (0.6–5.8)	1.5 (0.3–2.3)	0.3146	–
D_1_(skin, %)	19.8 (8.9–26.8)	21.8 (13.2–38.0)	17.1 (4.2–25.7)	*0.0425*	HIPO-IPSA: 0.0485 IPSA-FO: 0.0210
D_0.1_(lung, %)	42.7 (8.5–64.0)	57.4 (8.0–68.7)	44.7 (25.0–74.0)	*0.0457*	HIPO-IPSA: 0.0024 IPSA-FO: 0.0083
D_0.1_(heart, %)	22.7 (7.5–49.2)	23.7 (6.1–55.6)	22.9 (8.0–41.0)	0.8984	–

*V100, V150* the volume of the PTV (planning target volume) receiving 100% and 150% of the prescribed dose (%), *V*_*PTV*_ the volume of the PTV, *DNR* dose nonuniformity ratio, *COIN* conformal index, *V50(non-target breast)* absolute volume of the non-target breast irradiated by 50% of the prescribed dose (cc), *D*_*1*_*(contralateral breast), D*_*1*_*(skin)* the minimal dose of the most exposed 1 cc of the contralateral breast and skin (%), *D*_*0.1*_*(lung), D*_*0.1*_*(heart)* the minimal dose of the most exposed 0.1 cc of lung and heart (%)

^a^median dose–volume parameters

^b^Friedman ANOVA and LSD post-hoc test (italicized *p*‑values are significant)

**Table 2:** Dose–volume parameters (mean and range, median) in interstitial cervical BT (brachytherapy) using HIPO (hybrid inverse planning optimisation), IPSA (inverse planning simulated annealing) and forward optimisation (FO)

Dose–volume parameter	HIPO	IPSA	FO	*p*-value (ANOVA)^b^	*p*-value (post hoc)^b^
Nr. of needles	3 (0–6)^a^			–	–
V_PTV_ (cc)	35.6 (8.3–100.2)			–	–
V100 (%)	90.4 (83.2–95)	89.2 (78.9–95.4)	91.0 (84.4–95.6)	*0.0045*	HIPO-IPSA: 0.0352 IPSA-FO: 0.0214
V150 (%)	59.6 (44.8–67.3)	52.7 (25.8–66.4)	59.0 (44.2–71.4)	*<0.001*	HIPO-IPSA: 0.0053 IPSA-FO: 0.0009
V_100_ (cc)	48.5 (14.6–118.6)	59.6 (20.7–129.3)	52.8 (16.0–118.4)	*<0.001*	HIPO-IPSA: 0.0015 IPSA-FO: 0.0186
V_150_ (cc)	26.7 (7.8–68.2)	30.3 (7.4–73.9)	28.9 (8.5–65.8)	*<0.001*	HIPO-IPSA: 0.0017 IPSA-FO: 0.0498
DNR	0.55 (0.44–0.58)	0.50 (0.36–0.57)	0.54 (0.44–0.58)	*<0.001*	HIPO-IPSA: 0.0035 IPSA-FO: 0.0023
COIN	0.60 (0.30–0.73)	0.47 (0.22–0.66)	0.58 (0.34–0.87)	*<0.001*	HIPO-IPSA: 0.0033 IPSA-FO: 0.0043
D_2_(bladder, Gy)	4.1 (1.5–7.6)	4.3 (1.6–7.7)	4.1 (1.5–7.9)	0.2908	–
D_2_(rectum, Gy)	2.5 (0.6–6.3)	2.7 (0.6–7.2)	2.6 (0.6–7.9)	*0.0009*	HIPO-IPSA: 0.0024 IPSA-FO: 0.0083
D_2_(sigmoid, Gy)	3.2 (1.2–4.5)	3.6 (1.8–6.1)	3.1 (2.0–5.6)	*<0.001*	HIPO-IPSA: 0.0284 IPSA-FO: 0.0059
D_2_(bowel, Gy)	4.1 (1.8–6.2)	4.6 (2.9–7.4)	4.2 (2.5–5.5)	*0.0049*	HIPO-IPSA: 0.0301 IPSA-FO: 0.0412

*V100, V150* the volume of the PTV (planning target volume) receiving 100% and 150% of the prescribed dose (%), *V*_*100*_*, V*_*150*_*, V*_*PTV*_ absolute volume irradiated by 100% and 150% of the prescribed dose (cc) and the volume of the PTV, *DNR* dose nonuniformity ratio, *COIN* conformal index, *D*_*2*_*(bladder), D*_*2*_*(rectum), D*_*2*_*(sigmoid), D*_*2*_*(bowel)* the minimal dose of the most exposed 2 cc of the bladder, rectum, sigmoid and bowel (Gy)

^a^median dose-volume parameters

^b^Friedman ANOVA and LSD post-hoc test (italicized *p*‑values are significant)

**Table 3:** Dose–volume parameters (mean and range, median) in HDR (high-dose-rate) interstitial prostate BT (brachytherapy) using HIPO (hybrid inverse planning optimisation), IPSA (inverse planning simulated annealing) and forward optimisation (FO)

Dose–volume parameter	HIPO	IPSA	FO	*p*-value (ANOVA)^b^	*p*-value (post hoc)^b^
Nr. of needles	18 (16–20)^a^			–	–
V_PTV_ (cc)	39.5 (20.1–74.0)			–	–
V100 (%)	97.1 (89.0–99.0)	96.2 (94.8–98.5)	97.7 (97.0–98.5)	*0.0005*	HIPO-IPSA: 0.0466 IPSA-FO: 0.0237
V150 (%)	30.1 (22.1–37.0)	38.0 (30.5–56.3)	38.7 (22.0–59.9)	*<0.001*	HIPO-IPSA: 0.0011 HIPO-FO: 0.0008
DHI	0.70 (0.61–0.82)	0.60 (0.44–0.73)	0.61 (0.38–0.77)	*<0.001*	HIPO-IPSA: 0.0024 HIPO-FO: 0.0016
COIN	0.82 (0.73–0.91)	0.70 (0.61–0.72)	0.70 (0.42–0.74)	*<0.001*	HIPO-IPSA: 0.0008 HIPO-FO: 0.0003
D_0.1_(urethra, %)	113.8 (107.0–119.1)	112.6 (107.4–123.9)	124.6 (113.7–140.3)	*<0.001*	HIPO-FO: 0.0046 IPSA-FO: 0.0022
D_2_(rectum, %)	57.4 (46.2–91.0)	59.2 (52.4–72.2)	50.5 (34.8–59.4)	*<0.001*	HIPO-FO: 0.0057 IPSA-FO: 0.0011

*V100, V150* the volume of the PTV (planning target volume) receiving 100% and 150% of the prescribed dose (%), *V*_*PTV*_ the volume of the PTV, *DHI* dose homogeneity index, *COIN* conformal index, *D*_*0.1*_*(urethra)* the minimal dose of the most exposed 0.1 cc of the urethra (%), *D*_*2*_*(rectum)* the minimal dose of the most exposed 2 cc of the rectum (%)

^a^median dose–volume parameters

^b^Friedman ANOVA and LSD post-hoc test (italicized *p*‑values are significant)

## Discussion

In several types of cancer, image-guided interstitial brachytherapy has no better alternative at present, including high-tech EBRT as volumetric modulated arc therapy or stereotactic radiation therapy [3]. In spite of the fact that inverse dose optimisation is one of the hot topics in EBRT, its role in BT planning has not been evaluated systematically.

Choi et al. compared HIPO and IPSA algorithms for HDR interstitial tongue BT [6]. They found that these methods generate similar dosimetric results; however, the total dwell time with IPSA is 4 s longer than that of HIPO. They used 4–8 needles. Graphical optimisation was needed for the target coverages to satisfy the clinical goal. Then, the total dwell time was increased by approximately 10%.

We found superfluous active lengths in interstitial breast BT cases using the IPSA algorithm. Additionally, despite that the contour of PTV was not a concave shape, there were inactive dwell positions between two active positions. The coverage of the PTV with the prescribed dose was significantly lower with IPSA (D90: 101%) than with HIPO (102%) or FO (102.7%), but this difference is not important clinically. The volumes irradiated by a high dose (V150) were larger with HIPO (35.5%) and FO (36.0%) than with IPSA (33.2%), but the conformality was higher with HIPO and IPSA than with FO (COIN: 0.72, 0.71, 0.69, respectively). Nevertheless, forward-optimised plans were as homogeneous as inverse plans, DNR was 0.30 with HIPO, 0.30 using the IPSA algorithm and 0.29 with FO. The dose to the skin and lung was significantly lower using HIPO and FO compared to the IPSA method. Overall, FO and HIPO generated dosimetrically acceptable treatment plans in interstitial breast BT. If all the necessary volumes of interest are available, HIPO reduces the overall planning time.

**Fig. 3:**
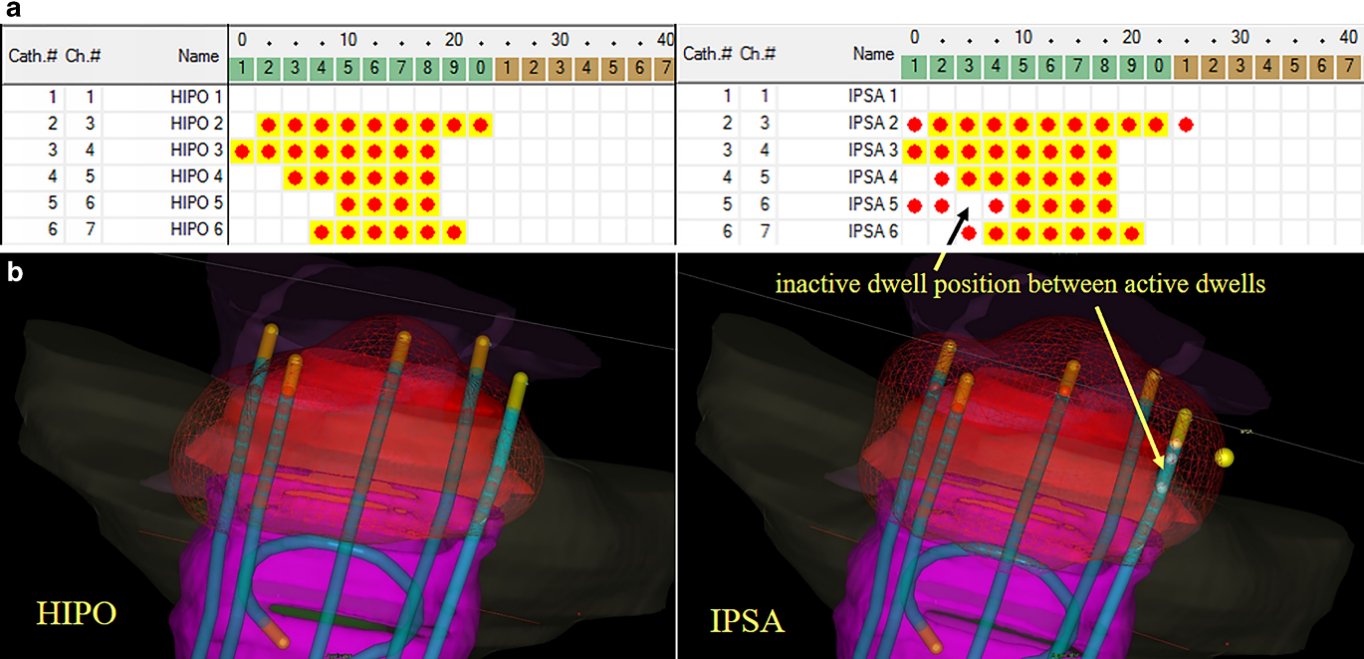
The active dwell positions in the intracavitary applicator and in the interstitial needles in the case of HIPO (hybrid inverse planning optimisation) and IPSA (inverse planning simulated annealing) optimisation methods. **a** *Red dots:* active dwell positions, *red dots with yellow background: *active dwell positions inside the target volume. **b** *Red dots:* active dwell positions, *blue:* the interstitial intracavitary applicator (volumes: *red:* PTV (planning target volume), *yellow:* bladder, *green: *rectum, *violet:* sigmoid, *pink:* vagina)

Thibault et al. investigated the clinical outcome of inverse-planned interstitial gynaecological BT [8]. They used a perineal template for implantation of a median of 17 needles and the IPSA algorithm for dose optimisation. They found that the D90 parameter correlates with local tumour control. Matias et al. compared the dosimetric results of FO and HIPO and found that the two methods are dosimetrically comparable [26]. Trnková et al. compared forward and inverse interstitial cervical BT plans using the obsolete Plato planning system for FO and the special gynaecologic treatment planning system Oncentra Gyn for inverse planning [9]. They stated that the conformality is the highest using HIPO, and the treatment time is less than in FO and IPSA. IPSA tends to overload the needles and needs additional contours to work. Our present work shows that IPSA generated lower-quality treatment plans than FO and HIPO methods in all the examined dosimetric parameters. IPSA resulted in longer active lengths and inactive dwell positions between active ones, as can be seen in Fig. 3. It resulted in significantly larger volumes irradiated by the prescribed dose (Fig. 2) and high-dose volumes (V_150_). In the optimal case, isodose surface volume correlates with the volume of the PTV [27]. FO and HIPO generated clinically similar dosimetric results, but HIPO needs additional adjustment with FO to reach the dose–volume constraints, especially in the case of a small number of needles. The number of needles correlates with the dosimetric quality of the treatment plan, as we showed in our previous study [11].

Pokharel et al. evaluated the HIPO algorithm in HDR prostate BT [28]. They found that HIPO can provide treatment plans with comparable target coverage to that of FO with a reduction in dose to the critical structures; however, HIPO resulted lower target coverage compared to FO. Panettieri et al. compared IPSA and HIPO [10] and stated that IPSA generates large dwell times in particular positions of the catheter, which can be the cause of the resultant lower homogeneity compared to the HIPO method. Poulin et al. made a comparison of optimisation algorithms in prostate BT and found that dose optimisation engines give similar dosimetric results [29]. Dinkla et al. made a comparison between graphical, IPSA and HIPO optimisation methods in HDR/PDR prostate BT [7]. They found that dose–volume parameters are comparable for all methods, and inverse algorithms resulted in shorter planning time than graphical optimisation (6.7 vs. 7.6 min, on average). We also experienced reduction of the optimisation time with HIPO and IPSA methods, but the effectiveness of IPSA was suboptimal: it generated dosimetrically acceptable plans, but the value of all the dose–volume parameters was inferior to using FO or HIPO methods. Additionally, IPSA created superfluous active lengths outside the prostate besides the underdosed prostate region close to rectum. FO and HIPO resulted in dosimetrically similar plans, but the PTV dose coverage was higher using FO (D90: 112.2% with FO vs. 110.4% with HIPO), the high-dose volumes were smaller with HIPO (V150: 30.1% with HIPO vs. 38.7% with FO), and HIPO was also more homogeneous (DHI: 0.7 vs. 0.6) and conformal (COIN: 0.8 vs. 0.7, respectively). The dose to the urethra was lower with HIPO (D_0.1_: 113.8% vs. 124.6%), but the rectal dose was higher (D_2_: 57.4% vs. 50.5%).

Taking every result into account, the IPSA optimisation method resulted in suboptimal treatment plans and used unnecessarily longer active lengths in interstitial breast, cervical and prostate BT (this superfluous length is usually 1 or 2 dwell positions, so 2.5–5 mm). Using the HIPO algorithm, active dwell positions can be determined before the optimisation of the dwell times in postimplant planning (in breast and cervical BT), and there is an option to plan the needles and active dwells inversely during live planning (in prostate BT) based on our predefined rules. Additionally, with the dwell time gradient restriction option, HIPO can produce homogeneous dwell time distribution. In HDR interstitial breast and prostate BT, HIPO can be recommended for dose optimisation; however, FO also results in dosimetrically acceptable plans, but with longer planning time. In the case of HDR interstitial cervical BT, sometimes a small number of needles does not give enough opportunities for inverse optimisation. To achieve the recommended dose coverage of PTV, additional FO is needed. With FO, isodoses can be expanded in the areas where organs at risk are not close to the PTV, while they can be decreased near to these tissues.

## Conclusion

In HDR interstitial breast and prostate BT, HIPO results in comparable dose–volume parameters to FO, but HIPO plans are more conformal. FO needed more planning time and more experience of the physicist. In cervical BT, HIPO produces dosimetrically acceptable plans only if a larger number of needles are used, and in this case the combination of FO and HIPO is recommended. The dosimetric quality of IPSA plans is suboptimal and results in unnecessarily larger active lengths.

## Appendix

**Table 4:** Initial dose–volume constraints for the HIPO optimisation algorithms in interstitial breast, cervical and prostate BT

HIPO	Region of interest	Min value (%)	Min weight	Max value (%)	Max weight
Breast	PTV	100	75	150	25
	Skin	–	–	50	40
	Ribs	–	–	50	30
	Normal tissue	–	–	120	5
Cervix	HR-CTV	100	80	150	5
	Bladder	–	–	70	10
	Rectum	–	–	50	10
	Sigmoid	–	–	60	10
	Normal tissue	–	–	120	5
Prostate	Prostate	100	70	150	5
	Urethra	–	–	120	40
	Rectum	–	–	50	20
	Normal tissue	–	–	120	8

**Table 5:** Initial dose–volume constraints for the IPSA optimisation algorithms in interstitial breast, cervical and prostate BT

		Surface				Volume				
IPSA	Region of interest	Min value (%)	Min weight	Max value (%)	Max weight		Min value (%)	Min weight	Max value (%)	Max weight
Breast	PTV	100	100	150	1		100	100	150	1
	Skin	–	–	40	40		–	–	–	–
	Ribs	–	–	50	30		–	–	–	–
	Non-target breast	–	–	120	5		–	–	35	100
Cervix	HR-CTV	200	100	150	15		200	100	150	10
	Bladder	–	–	70	15		–	–	70	15
	Rectum	–	–	50	15		–	–	50	15
	Sigmoid	–	–	60	15		–	–	60	15
Prostate	Prostate	100	5	150	1		100	5	200	0.1
	Urethra	100	1	108	3		100	1	108	3
	Rectum	–	–	60	3		–	–	60	3
